# Photosynthetic limitation as a factor influencing yield in highbush blueberries (*Vaccinium corymbosum*) grown in a northern European environment

**DOI:** 10.1093/jxb/ery118

**Published:** 2018-03-24

**Authors:** Antonios Petridis, Jeroen van der Kaay, Elina Chrysanthou, Susan McCallum, Julie Graham, Robert D Hancock

**Affiliations:** Cell and Molecular Sciences, The James Hutton Institute, Invergowrie, Dundee, UK

**Keywords:** ^13^CO_2_-labelling, carbon assimilation, carbon storage, sink tissues, starch, *Vaccinium*, *corymbosum*, yield

## Abstract

Published evidence indicates that nearly 60% of blueberry-producing countries experience yield instability. Yield is a complex trait determined by genetic and environmental factors. Here, using physiological and biochemical approaches, we tested the hypothesis that yield instability results from year-to-year environmental variation that limits carbon assimilation, storage and partitioning. The data indicate that fruit development depends primarily on the daily production of non-structural carbohydrates by leaves, and there is no accumulation of a starch buffer to allow continuous ripening under conditions limiting for photosynthesis. Photosynthesis was saturated at moderate light irradiance and this was mainly due to stomatal and biochemical limitations. In a dynamic light environment, photosynthesis was further limited by slow stomatal response to increasing light. Finally, labelling with ^13^CO_2_ at specific stages of fruit development revealed a relatively even distribution of newly assimilated carbon between stems, roots and fruits, suggesting that the fruit is not a strong sink. We conclude that a significant component of yield variability results from limitations in photosynthetic efficiency that are compounded by an inability to accumulate starch reserves in blueberry storage tissues in a typical northern European environment. This work informs techniques for improving agronomic management and indicates key traits required for yield stability in such environments.

## Teaser

Photosynthetic limitation under a northern European environment precludes carbon storage and drives fruit abortion in blueberries causing yield instability. This highlights the need to develop cultivars adapted to maritime environments.

## Introduction

In fruit trees, photosynthesis and whole plant carbohydrate partitioning impact greatly on yield, and any factor that disrupts these processes may lead to fruit abortion and hence yield losses (Zhang *et al.*, 2005; Teo *et al.*, 2006; Gauci *et al.*, 2009; Osorio *et al.*, 2014). Yield-limiting factors may be related to adverse environmental conditions and insufficient pollen load (external factors), or to reduced carbohydrate reserves that result in competition between sink organs (internal factors) (Gauci *et al.*, 2009). The problem of sink competition due to resource limitation could be exacerbated if critical developmental stages of different sinks coincide, as is often the case with developing fruits and vegetative growth (Dal Cin *et al.*, 2009). Lack of carbohydrate resources can result from limitations in carbon fixation and assimilation. These limitations may be further compounded if a plant is unable to accumulate carbohydrate reserves that can be utilized to support continued fruit development under conditions limiting for photosynthesis.

Environmental factors significantly influence photosynthesis and carbohydrate partitioning. For example, photosynthesis varies widely in response to light intensity and temperature over time and among different environments. Most species increase their photosynthetic capacity in environments with high *versus* limiting light availability, and mild *versus* extreme temperatures (Demmig-Adams *et al.*, 2017). Furthermore, environmental factors such as temperature have significant influence on carbon partitioning between different organs (Hancock *et al.*, 2014; Pastenes *et al.*, 2014; Salazar-Parra *et al.*, 2015). In addition to environmental effects, photosynthetic capacity and carbohydrate partitioning are influenced by the competitive ability of a sink organ to import photoassimilates – a characteristic known as sink strength (Herbers and Sonnewald, 1998). Such regulation of photosynthesis by demand involves the activation of photosynthetic genes and is orchestrated by signals associated with the balance between carbohydrate production in leaves and its utilization in sink tissues (Foyer and Paul, 2001; Demmig-Adams *et al.*, 2017). Generally, high sink strength enhances photosynthesis, whereas low sink strength leads to higher carbohydrate accumulation in leaves and subsequent feedback downregulation of photosynthesis (Demmig-Adams *et al.*, 2017).

Over the past 20 years, the production of highbush blueberry (*Vaccinium corymbosum* L.) has expanded worldwide. The majority of the countries that produce blueberries use cultivars that were initially bred in the USA (Retamales and Hancock, 2012). There is substantial evidence indicating that many of these countries experience significant instability in blueberry yield (FAO, 2014; Supplementary Fig. S1 at *JXB* online); however, the reasons for yield instability have not been previously investigated. On one hand, yield instability could be due to sudden and largely unpredictable catastrophic events that occur during vulnerable stages of fruit development. For example, the late spring frosts that occurred in the eastern and mid-western regions of the United States in 2007 and 2012, when plants were at the flowering or green-fruit stage, caused significant damage to blueberry production (Warmund *et al.*, 2008; Rowland *et al.*, 2013), although due to the large geographic range of blueberry production in the USA these localized events had little impact on country-wide yield (Supplementary Fig. S1). On the other hand, yield instability could arise from subtle year-to-year environmental variation that disrupts carbon assimilation, storage, and partitioning.

Here, we examined, by means of physiological and biochemical approaches, the processes underlying yield in blueberries. While our ultimate goal is to develop cultivars that produce high and stable yields in northern European maritime environments, we first needed to establish the mechanisms underlying the yield instability trait in blueberries. We conclude that a significant component of yield instability results from limitations in photosynthetic efficiency and is compounded by limited accumulation of starch reserves in storage tissues. Our work additionally informs techniques for improving agronomic management practices and for developing robust genetic markers to accelerate breeding of yield-stable blueberry germplasm.

## Materials and methods

### Plant material, growth conditions, and plant harvest

Most of the experiments were carried out at the experimental site of the James Hutton Institute (Dundee, Scotland, UK, 56°27ʹ25ʹʹN, 3°4ʹ11ʹʹW) during the 2016 and in some instances during the 2017 growing seasons, while one experiment, involving the use of reflective mulches, was conducted at a commercial site (Castleton Farm Ltd, Scotland, UK, 56°53ʹ56ʹʹN, 2°23ʹ42ʹʹW) during the 2017 growing season. For the experiments at the James Hutton Institute, 3-year-old blueberry plants (*Vaccinium corymbosum* L. ‘Liberty’ and ‘Duke’) were grown outdoors in 10 litre pots filled with substrate (Levington Advance CNS Ericaceous, pH 4.4–5.0; ICL, Ipswich, UK).

The experiment with reflective mulches was conducted using 9-year-old *V. corymbosum* ‘Liberty’ plants grown in the soil under a polytunnel. Reflective mulches were placed either side of the row when plants were at the early green-fruit stage. Two types of reflective mulch were used to manipulate the light environment: (1) silver-coloured mulch (Diamond Lightite®, Easy Grow Ltd, Grimsby, UK) and (2) white-coloured mulch (White Lightite®, Easy Grow Ltd). The experimental design was a randomized block. Each block consisted of three treatments and three plants per treatment. The total number of blocks was three.

To examine sugar and starch distribution in blueberry organs, whole ‘Liberty’ and ‘Duke’ plants were harvested at the James Hutton Institute site at specific stages of development: bud dormancy, bud swell, shoot expansion, green fruit, fruit colouring, ripening fruit, and post-harvest. Green fruit, ripening and post-harvest stages were also selected for ^13^C labelling experiments. Sugar and starch distribution in blueberry organs was examined during the 2016 and 2017 growing seasons. At each harvest date, three replicate plants of each cultivar were transferred to the laboratory and the above ground organs (leaves, fruits or buds, current-year shoots, and old wood stems) were separated and immediately frozen in liquid N_2_. Roots were washed to remove soil and then placed in liquid N_2_. Following snap freezing, samples were stored for a maximum of 2 d at −20 °C until freeze-drying. Frozen samples were freeze dried (Martin Christ Gefriertrocknungsanlagen GmbH, Osterode, Germany), milled to a fine powder (Cyclone Mill Twister, Retsch, Haan, Germany) and stored at −20 °C until analysis.

Environmental parameters were recorded hourly using data loggers (Delta-T Devices Ltd, Cambridge, UK) placed outdoors or inside tunnels in the proximity of selected plants. Supplementary Figs S2, S3 show the photosynthetic photon flux density (PPFD) and mean air temperature for the 2016 growing season, respectively.

### Determination of sugars and starch

Sugars and starch were extracted as described by Viola *et al.* (2007). Fifty milligrams of lyophilized plant tissue were homogenized with 1 ml of 80% ethanol and incubated at 80 °C for 1 h with periodic vortexing. The extracts were centrifuged at 16000 *g* for 10 min at 1 °C. After centrifugation, the supernatant was transferred into a 2 ml microcentrifuge tube and the extraction repeated. The two supernatants were combined, vacuum concentrated at 50 °C and lyophilized overnight. Samples were resuspended in 1 ml of sterile distilled H_2_O and centrifuged at 16000 *g* for 5 min at 1 °C. This fraction was used for the quantification of sugars using high performance anion exchange chromatography (HPAEC) as previously described (Nwankno *et al.*, 2012). The remaining pellets were washed twice with 1 ml distilled H_2_O, resuspended in 450 µl distilled H_2_O and starch was gelatinized by incubating samples at 100 °C for 2 h with periodic vortexing. The suspensions were cooled to room temperature and 50 µl of 1 M sodium acetate buffer (pH 5.2) containing 100 U ml^−1^ of α-amyloglucosidase (Sigma-Aldrich) was added. The samples were further incubated at 37 °C for 18 h to allow full conversion of starch to glucose and centrifuged at 16000 *g* for 5 min at 1 °C. Following centrifugation, the samples were stored at −20 °C until HPAEC analysis.

### Photosynthetic gas exchange and chlorophyll fluorescence measurements

Gas exchange and chlorophyll fluorescence measurements were conducted on the fourth, fully expanded leaf of ‘Liberty’ shoots selected on the basis of uniformity. All measurements were conducted on three independent replicate plants. Net carbon assimilation rate (*A*), stomatal conductance (*g*_s_), and intercellular CO_2_ concentration (*C*_i_) were measured using a CIRAS-2 portable photosynthesis system (PP Systems, Amesbury, MA, USA) equipped with a PLC6 (U) 2.5 cm^2^ leaf cuvette, which provided light through an integrated LED light unit. Leaf temperature was maintained at 25 °C and relative humidity at 60%. With the exception of *A*–*C*_i_ curves, CO_2_ concentration was supplied at 400 µmol mol^−1^ with a gas flow rate of 200 ml min^−1^.

To generate light response curves, leaves of ‘Liberty’ were acclimated at 2000 µmol m^−2^ s^−1^ PPFD for 50 min. After acclimation, light irradiance was decreased using the following PPFD sequence: 2000, 1600, 1000, 600, 400, 300, 200, 150, 100, 50, and 0 µmol m^−2^ s^−1^. Before recording gas exchange parameters, leaves were equilibrated at each irradiance for 4 min. Light response curves were generated at 25 °C and 15 °C and the same leaf was used for both temperatures; 25 °C was chosen as representing a high temperature for the UK growing season, while 15 °C was chosen as representing more moderate temperature.

*A*–*C*_i_ curves were generated on ‘Liberty’ and ‘Duke’ leaves at 1200 µmol m^−2^ s^−1^ using the following stepwise gradients: 400, 200,100, 50, 400, 500, 600, 800, 1000, 1200, 1400, and 1600 µmol mol^−1^.

The response of *A* and *g*_s_ to changing irradiance was measured on ‘Liberty’ and ‘Duke’ plants according to McAusland *et al.* (2016), except that readings were recorded every 3 min. Briefly, leaves were acclimated at 100 µmol m^−2^ s^−1^ PPFD for 45 min, until both *A* and *g*_s_ reached ‘steady state’ levels. PPFD was then increased to 1000 µmol m^−2^ s^−1^ for 1 h, before returning to 100 µmol m^−2^ s^−1^ for 30 min.

Chlorophyll fluorescence parameters were measured concomitantly with light response curves using the chlorophyll fluorescence module (CFM) of CIRAS-2. Prior to generating light response curves, leaves were dark-adapted for 40 min. Then, a saturating red-light pulse (0.8 s; 6000 µmol m^−2^ s^−1^) was applied to determine the maximum quantum yield of photosystem II. Following acclimation at 2000 µmol m^−2^ s^−1^ for 45 min (as described above), gas exchange parameters and electron transport rate were recorded simultaneously.

### ^13^C isotope labelling

Labelling experiments with ^13^CO_2_ were performed on the same *V. corymbosum* ‘Liberty’ plants used for sugar and starch analysis during the green-fruit (2016, 2017), ripening fruit (2016, 2017), and post-harvest (20 d after harvest, 2016) stages. The latter stage was examined only in 2016, because in 2017 leaves were already senescent at the post-harvest stage and therefore CO_2_ uptake was not expected to be efficient. All labelling experiments started at 09.00 h.

Whole plants were enclosed in transparent plastic bags and labelled with ^13^CO_2_ for 1 h. To generate ^13^CO_2_, 3 ml of 70% lactic acid was injected into glass vials, containing 1 g of NaH^13^CO_3_ (^13^CO_2_, treated plants) or NaH^12^CO_3_ (^12^CO_2_, control plants). The glass vials were mounted on the pots before covering the plants with the bags. When ^13^CO_2_ labelling ended, the bags were removed and ^13^C was chased for 4 and 24 h before plant harvest.

The stable carbon isotopic composition (δ^13^C) and carbon content of lyophilized powdered material were analysed by OEA Laboratories Ltd (Callington, UK) using a dual-pumped Sercon 20-20 isotope ratio mass spectrometer (IRMS, Sercon Ltd, Crewe, UK) coupled to a Thermo EA1110 elemental analyser (Thermo Fisher Scientific, Waltham, MA, USA). For each sample, approximately 1.15–1.2 mg of tissue was weighed and four standards were used to calibrate the data (USGS40 l-glutamic acid, USGS41a l-glutamic acid, IAEA-CH6 sucrose and NBS1577b bovine liver). Excess δ^13^C (‰) of a given organ was calculated by subtracting δ^13^C values after 4 and 24 h of chasing from δ^13^C values of control plants.

### Statistical analysis

Data were subjected to one-way analysis of variance (ANOVA). Significant differences (*P*≤0.05) between means were determined using either Tukey’s test or Fisher’s least significant difference (LSD) test. Correlation between variables was achieved using Pearson’s correlation coefficient, considering a confidence level of 95% (*P*≤0.05). Statistical analysis was performed using IBM SPSS Statistics for Windows, Version 22.0 (IBM Corp., Armonk, NY, USA).

## Results

### Historic yield data and loss of reproductive structures

**Fig. 1.**
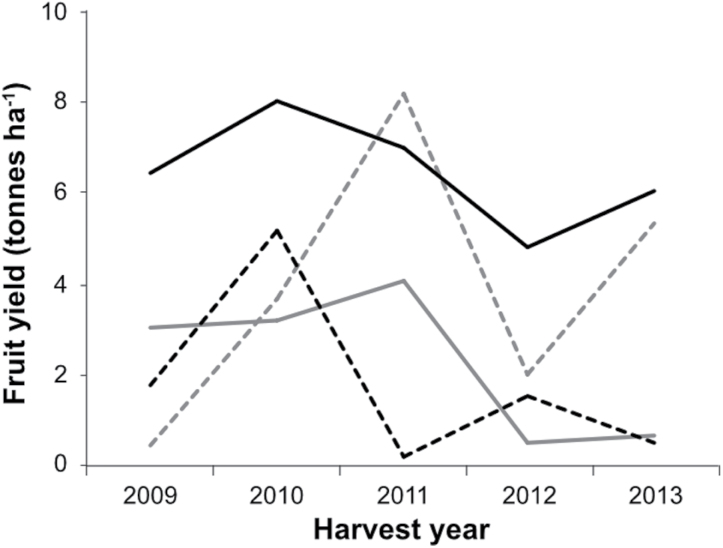
Reported yields of the blueberry cultivars ‘Chandler’ (black solid line), ‘Darrow’ (black dashed line), ‘Brigitta blue’ (grey solid line) and ‘Aurora’ (grey dashed line) grown between 2009 and 2013 under UK conditions in Blairgowrie, Scotland, UK.

Anecdotal evidence suggested that UK growers experience significant year to year yield instability in the blueberry crop. In order to substantiate this, we searched the database of the United Nations Food and Agriculture Organisation (FAO, 2014). Although no information was available regarding UK blueberry production, there were data for several other countries, some of which are major blueberry producers. Several countries exhibit significant yield instability (Supplementary Fig. S1). To gain further insight into the problem of yield instability in the UK, we sought historical yield data from specific growers. Figure 1 shows the yield of four blueberry cultivars (‘Aurora’, ‘Brigitta blue’, ‘Chandler’, and ‘Darrow’) grown at a commercial site in Scotland during the 2009–2013 growing seasons. All cultivars exhibited a high degree of yield variability without observing any particular trend that would otherwise point to the influence of a specific factor(s). Indeed, yield instability is independent of genotype, plant age, and growing year (Fig. 1), indicating that yield instability in blueberries is a complex phenomenon originating from an as yet unknown factor(s).

**Fig. 2.**
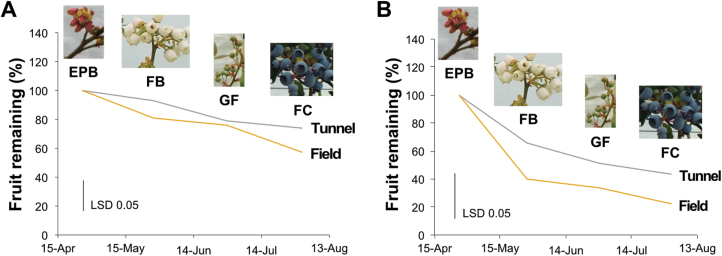
Relative change in the number of reproductive structures in ‘Liberty’ (A) and ‘Duke’ (B) plants (*n*=3) during the 2016 growing season at the Invergowrie site. Plants were grown either under protective polytunnels or in the open field and the number of reproductive structures were regularly counted at different stages of development as indicated (EPB, early pink bud; FB, full bloom; FC, fruit colouring; GF, green fruit). Data represent the mean of three independent plants and the LSD 0.05 value is indicated.

To determine whether there is any particular developmental stage that is vulnerable to loss of reproductive structures, we counted the number of buds, flowers, or fruits of selected shoots during specific stages of fruit development (Fig. 2). The scoring of reproductive-structure losses was conducted in ‘Liberty’ and ‘Duke’ plants grown under either open-field or tunnel conditions. For cultivar ‘Liberty’, abortion of reproductive structures occurred gradually during the entire growing season, regardless of growing environment (Fig. 2). Cultivar ‘Duke’ exhibited a similar trend, although a higher rate of fruit abortion was evident during the flowering stages. Similar results were also obtained during the 2017 growing season (Supplementary Fig. S4). Taken together, these results suggest that fruit loss is not due to an environmentally catastrophic event such as frosting of flowers, but occurs instead during all stages of fruit development.

### Changes in non-structural carbohydrate pools of different organs at specific stages of blueberry development

**Fig. 3.**
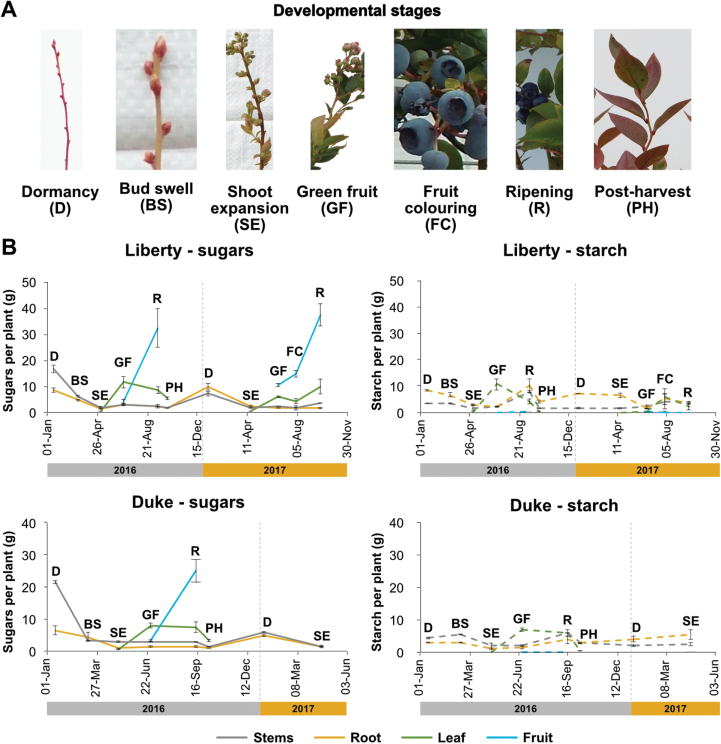
Changes in whole organ soluble sugars and starch throughout the annual plant growth cycle. (A) Pictorial indication of the specific stages of plant development that were selected for carbohydrate analysis. (B) Seasonal sugar and starch dynamics in different organs of ‘Liberty’ and ‘Duke’ plants as indicated (abbreviations defined in (A)). Vertical dotted lines separate 2016 and 2017 growing seasons. Data were normalized relative to a plant with 100 g DW old wood stem by dividing the mass of each organ in the plant by the mass of the stem and multiplying by 100. Each data point represents the mean value of three replicates ±SE. Different *x*-axis scales were used for ‘Liberty’ and ‘Duke’ in (B) to take account of the extended analysis period of ‘Liberty’ plants.

Although non-structural carbohydrates are major determinants of yield, little is known about their accumulation and distribution in blueberry plants. Additionally, there is no information regarding the dynamics of non-structural carbohydrates in different organs of blueberry plants during their annual cycle. Thus, to identify potentially critical periods for resource accumulation, sugar and starch dynamics were determined in different organs of ‘Liberty’ and ‘Duke’ plants (Fig. 3). Plants were harvested at specific stages of development including bud dormancy, bud swell, shoot expansion, green fruit, fruit colouring, fruit ripening, and post-harvest. Here, we present the sum of individual sugars (glucose, fructose, and sucrose) that make up the total soluble sugars in blueberry tissues. This provides information regarding the overall carbohydrate budget. Information regarding the quantity and fluctuation of individual sugars in different organs of blueberry plants throughout the growing season is provided in Supplementary Table S1.

Both cultivars exhibited similar behaviour with respect to the accumulation of soluble sugars and starch in different organs throughout the annual cycle (Fig. 3). During the cold winter months when buds were dormant, both stems and roots had the highest concentration of free sugars relative to other times of the year. The accumulation of sugars in the root was lower compared with sugar accumulation in stems. Starch reserves in roots and stems were also relatively high during this period. Both sugar and starch levels fell in storage tissues (roots and stems) as buds started to develop and shoots expanded. Indeed, carbohydrate reserves were almost completely utilized to support flowering and new leaf growth. As leaves became fully photosynthetically functional (green fruit stage), sugar and starch accumulation remained low in the storage tissues and in the immature fruit, but the leaves themselves represented the most significant reservoir of plant carbohydrates. As fruit ripened, there was a dramatic accumulation of sugars in the fruits, while leaf sugar content did not change. Additionally, at this stage there was a small increase in starch accumulation in the storage tissues. Finally, during the post-harvest stage sugar and starch content slightly decreased in each organ.

Taken together, these data indicate that unlike similar crops, such as blackcurrant (Hancock *et al.*, 2007), blueberry plants do not accumulate significant carbohydrate reserves at any point during development and fruit growth therefore appears to be supported from immediately assimilated carbon.

### Photosynthetic capacity is saturated at low light intensity and is restricted by photochemical, biochemical, and stomatal limitations

The analysis of sugar and starch dynamics demonstrates that blueberries do not accumulate a starch buffer in their storage tissues during fruit development. Consequently, fruit growth depends primarily on sugars produced in the leaves by photosynthesis. We therefore estimated CO_2_ fixation rate as a function of light intensity at fixed CO_2_ (light response curve) and as a function of CO_2_ concentration at a saturating light intensity (*A*–*C*_i_ curve) to determine the factors responsible for photosynthetic limitation.

**Fig. 4.**
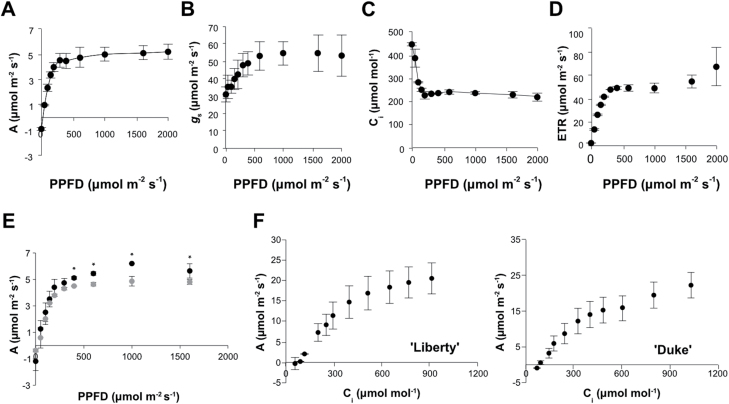
Blueberry leaf photosynthetic parameters estimated as a function of photosynthetic photon flux density and leaf internal CO_2_ concentration. Light-response curves of (A) net CO_2_ assimilation rate (*A*), (B) stomatal conductance to water vapour (*g*_s_), (C) internal CO_2_ concentration (*C*_i_), and (D) electron transport rate (ETR) measured on ‘Liberty’ plants. All measurements were undertaken on fully expanded leaves at 25 °C using a fixed cuvette CO_2_ concentration of 400 µmol mol^−1^. (E) Light response curve of *A* at 25 °C (black circles) and 15 °C (grey circles). Data were subjected to one-way ANOVA and significant differences (*P*≤0.05) between means were determined using Tukey’s test. Asterisks above data points indicate significant differences between 25 °C and 15 °C for the same light irradiance level. (F) *A*–*C*_i_ curves generated in ‘Liberty’ and ‘Duke’ plants using a photosynthetic photon flux density (PPFD) of 1200 µmol m^−2^ s^−1^ and leaf temperature of 25 °C. In all plots data are represented as mean ±SE, *n*=3.

First, we determined the effect of light on blueberry leaf photosynthesis by generating light response curves at 25 °C (Fig. 4A). Carbon assimilation rate (*A*) increased with increasing light irradiance, but was saturated at relatively low light intensity (400–500 µmol m^−2^ s^−1^ PPFD). Similar results were observed using fully expanded leaves in both the 2016 (Fig. 4A) and 2017 (Supplementary Fig. S5) growing seasons. Stomatal conductance (*g*_s_) increased with increasing light irradiance up to an irradiance of approximately 600 µmol m^−2^ s^−1^ after which no further increase was observed (Fig. 4B). Leaf internal CO_2_ concentration (*C*_i_) decreased with increasing light irradiance, up to a point where photosynthesis was saturated (Fig. 4C). To examine the effect of high irradiance on photochemistry, we measured electron transport rate (ETR), which provides information about the overall photosynthetic capacity *in vivo* (Genty *et al.*, 1989; Maxwell & Johnson, 2000). The results showed that electron transport rate increased almost linearly at low light irradiances and reached a plateau at 400–500 µmol m^−2^ s^−1^ PPFD (Fig. 4D). Correlation analysis between ETR and *A* or between ETR and *C*_i_ indicated a high positive or negative relationship, respectively (*r*=0.78 and *r*=−0.85, *P*≤0.01). At 15 °C, the response of carbon assimilation rate to increasing light irradiance was similar to that at 25 °C, but maximal assimilation rates were lower and there was a trend of lower assimilation at all light intensities, suggesting that photosynthesis was limited by biochemical processes at the lower temperature (Fig. 4E).

We further investigated the photosynthetic responses of blueberry leaves to increasing CO_2_ concentrations in ‘Liberty’ and ‘Duke’ leaves under an irradiance of 1200 µmol m^−2^ s^−1^ (Fig. 4F), a value significantly greater than that at which photoassimilation was saturated under ambient CO_2_ (Fig. 4A). In both cultivars, carbon assimilation rate increased continuously with increasing leaf CO_2_ concentration. These data again indicate biochemical limitation, suggesting that at ambient CO_2_ levels, carboxylation efficiency of ribulose 1,5-bisphosphate carboxylase oxygenase (Rubisco) limits photosynthetic assimilation rate.

**Fig. 5.**
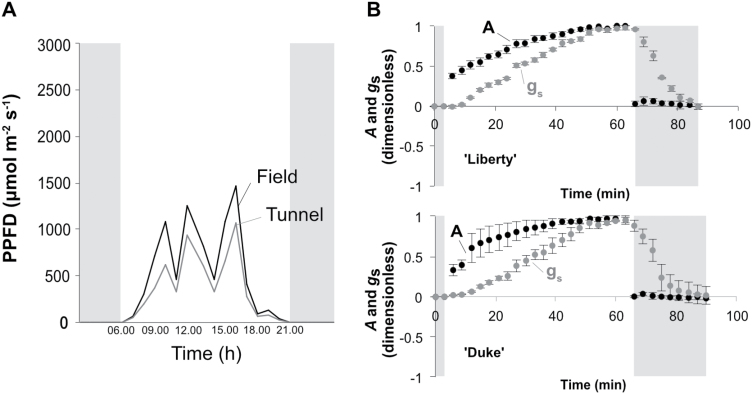
Responses of photosynthetic assimilation and stomatal conductance to a changing light environment in ‘Liberty’ and ‘Duke’ plants. (A) A representative example of daily changes in photosynthetic photon flux density (PPFD) recorded hourly at the Invergowrie site. (B) Normalized temporal responses of net CO_2_ assimilation rate (*A*) and stomatal conductance to water vapour (*g*_s_) to changing light. Leaves were equilibrated at 100 µmol m^−2^ s^−1^ PPFD for 30–45 min and once steady state was satisfied, PPFD was increased to 1000 µmol m^−2^ s^−1^ for 1 h before returning to 100 µmol m^−2^ s^−1^ for 30 min. *A* and *g*_s_ were recorded every 3 min and data are represented on a relative scale indicating the mean response ±SE of three independent replicates. Shaded areas on the plots indicate the pre-equilibration and recovery under low light while the unshaded area represents the period leaves were exposed to high light.

We also examined the photosynthetic responses of blueberry leaves to fluctuating light irradiance, typically observed under UK climatic conditions (Fig. 5A). To mimic this dynamic light environment, light intensity was changed from low (100 µmol m^−2^ s^−1^ PPFD) to high (1000 µmol m^−2^ s^−1^ PPFD) and *A* and *g*_s_ were recorded every 3 min. At the beginning of the change, *A* increased abruptly compared with *g*_s_ in both ‘Liberty’ and ‘Duke’ (Fig. 5B). Following this initial period, the increase in *A* slowed to a degree that was similar to the increase in *g*_s_, until reaching a point where *A* and *g*_s_ were steady and coupled. The time required for *A* and *g*_s_ to reach steady state levels was around 50 min for both cultivars. These data indicate that under a dynamic light environment, the slow speed of the stomatal response may lead to a stomatal limitation to photosynthesis.

Light response curves generated at 15 °C and 25 °C showed that photosynthesis is saturated at moderate light irradiance (~500 µmol m^−2^ s^−1^; Fig. 4A, E) and is attenuated at lower temperatures. To obtain detailed information about these critical environmental parameters influencing blueberry production, incident solar irradiance (PPFD) and air temperature were recorded inside and outside a horticultural polytunnel during flowering and fruit growth periods (1 May–30 September). Interestingly, 60% and 73% of the PPFD measured outside and inside the tunnel, respectively, from May to September was below 500 µmol m^−2^ s^−1^ (excluding night hours; Supplementary Fig. S2), suggesting that during the greater part of the growing season blueberry plants do not photosynthesize at their full potential. These data indicate that under UK growing environments, light levels fluctuate significantly around the value required for photosynthesis to operate at optimal levels in blueberry leaves and differences in light levels between seasons may explain blueberry yield instability. Mean daily temperature fluctuated between approximately 7 and 20 °C over the growing season (Supplementary Fig. S3). Data obtained from light response curves at two temperatures (Fig. 4E) indicate that this temperature range significantly affects photosynthetic assimilation rates and highlights seasonal variation in air temperature as a further factor likely to promote seasonal yield instability.

### Partitioning of newly assimilated carbon (^13^C) during fruit development

Having established that fruit yields rely on newly acquired photoassimilates and that photoassimilation is limited by external environmental factors, experiments were conducted to understand the distribution of newly acquired photoassimilates between different plant organs. ‘Liberty’ plants were labelled with ^13^CO_2_ at three stages of fruit development, i.e. green fruit, ripening and post-harvest. After 4 h of chasing, the highest concentration of ^13^C was found in the leaves for all stages, while significant concentrations were also recovered in fruit, buds and current year shoots. The lowest concentrations of ^13^C were recovered in old wood stems and roots (Supplementary Fig. S6). After 24 h of chasing, ^13^C concentration decreased almost two-fold in leaves, but increased in fruits and to a lesser extent in current-year shoots. A small increase in ^13^C concentration was found in the roots after chasing for 24 h, but only in the last two stages. ^13^C concentration in old-wood stems after 24 h chasing was similar to that found after 4 h (Supplementary Fig. S6).

**Fig. 6.**
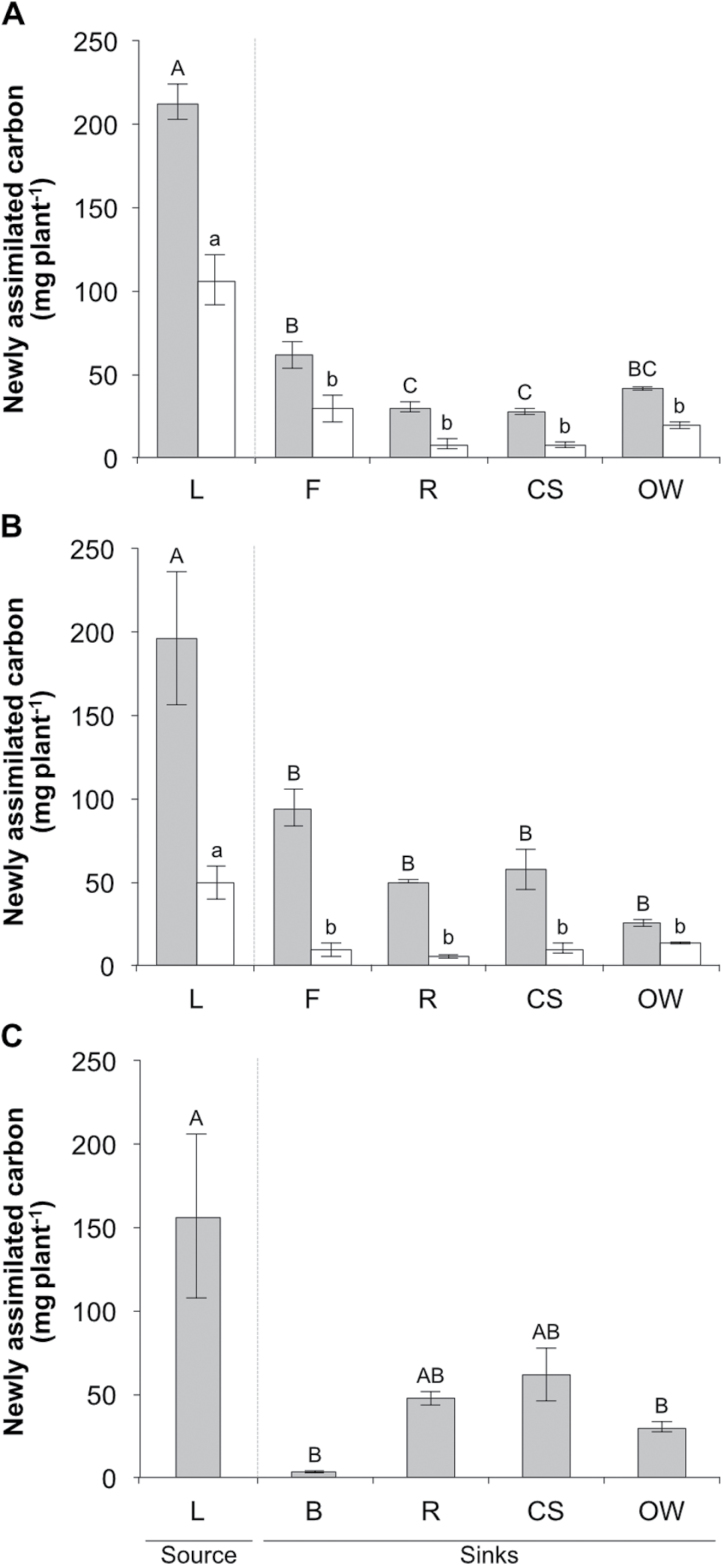
Newly assimilated carbon in individual organs of ‘Liberty’ during fruit set (A), ripening (B), and post-harvest (C). Plants were labelled for 1 h with ^13^CO_2_ and harvested after chasing for 24 h. ^13^C content was estimated in leaves (L), fruit (F), roots (R), current year shoots (CS), old wood stems (OW), and buds (B), and data were used to estimate the mean total ^13^C-assimilation in three replicate plants normalized to 100 g DW of old wood stems. Grey and white bars represent data from 2016 and 2017 growing seasons, respectively. Dotted lines separate source (leaf) from sink tissues. Bars are the mean value of three replicates±SE. Data were subjected to one-way ANOVA. Significant differences (*P*≤0.05) between means were determined using Tukey’s test. Uppercase and lowercase letters denote differences between tissues for 2016 and 2017, respectively.

When taking into consideration the mass of the different organs to estimate the relative distribution of newly assimilated carbon 24 h after a 1 h labelling period, at most stages in both years the majority of new assimilates remained associated with the leaves (Fig. 6), suggesting incomplete assimilate export after a single dark period. The relative strength of different plant sink tissues exhibited significant variation dependent on fruit developmental stage. During fruit set in 2016, significantly more newly assimilated carbon was allocated to fruit than to roots, current year shoots, and old wood stems, which all accumulated similar amounts of total assimilate (Fig. 6A). A similar pattern of assimilate distribution was observed in 2017 although in this year the amount of assimilate allocated to fruits was not significantly greater than other sink tissues (Fig. 6A).

At the fruit ripening stage and for both growing seasons, similar amounts of new photoassimilate were distributed to each of the sink tissues (Fig. 6B).

During the post-harvest stage very little photoassimilate was partitioned to buds in part as a result of the low total mass of the organs (Fig. 6C). At this stage in the plant growth cycle newly assimilated carbon was almost equally distributed between sink tissues.

Taken together, in conjunction with data indicating that blueberry plants do not accumulate significant carbohydrate stores (Fig. 3), these data suggest that plants balance their distribution of photoassimilate amongst all of the sink tissues and do not prioritize the development of fruit above the development of other plant organs.

### Effect of reflective mulches on blueberry yield

**Fig. 7.**
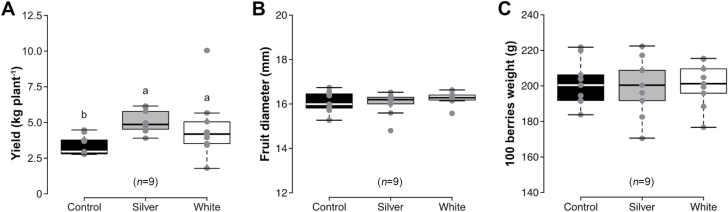
The influence of reflective mulches on total fruit yield and individual fruit size and weight. Total fruit yield (A), fruit diameter (B), and 100 berry weight (C) of cv. ‘Liberty’ plants grown over silver or white reflective mulches are indicated. Boxes comprise the interquartile range from the 25th to 75th percentile, horizontal lines within each box denote median values, and whiskers represent the range (*n*=9). Data were subjected to one-way ANOVA. Significant differences (*P*≤0.05) between means were determined using Tukey’s test. Boxes with different letters are significantly different from each other.

Gas exchange analysis (Fig. 4) and environmental data (Supplementary Fig. S2) indicate that yield instability in blueberries may be related to the variability in sunlight irradiance across different years where light may be more limiting in one year than another. To determine whether limiting light does indeed limit blueberry yield, experiments were conducted to improve light reflectance into the plant canopy using silver and white reflective mulches. Plants that were grown in the presence of reflective mulches throughout the growing season exhibited significant increases in fruit yield relative to those grown over bare ground (Fig. 7A). Plants grown over silver and white mulches increased their yield by 49% and 37%, respectively, compared with control plants. To determine whether growth over reflective mulches supported the maturation of more fruit or increased mass of the same number of fruits, fruit diameter and weight were estimated. The data support the hypothesis that growth over reflective mulches supports fruit retention, since neither fruit diameter (Fig. 7B) nor fruit weight (Fig. 7C) of mulch-treated plants differed from that of control plants.

## Discussion

Highbush blueberry (*Vaccinium corymbosum* L.) is a species native to North America and over the past 20 years its production has expanded worldwide. Most highbush blueberry cultivars currently grown in the UK and many other countries come from breeding programmes established in the USA and hence they are adapted to US climatic conditions. The adoption of US adapted cultivars for growth in the UK and other blueberry growing regions has occurred without any systematic assessment of the suitability of these cultivars for local climate and production systems with the consequence that significant yield instability is observed in many countries (Fig. 1; Supplementary Fig. S1). A key objective of the present study was therefore to determine the physiological and biochemical factors that resulted in yield instability under UK growing conditions.

### Blueberry fruit development is dependent on a ‘current carbon account’

To understand what factors might influence year-to-year variability in blueberry fruit yields, two hypotheses were considered. Firstly, yield instability could result from a catastrophic environmental event, such as frosting and loss of flowers shortly after bloom. Alternatively, more subtle environmental variations might limit the capacity of the plant to support fruit development. Analysis of the rate of loss of plant reproductive structures indicated that fruit loss occurred gradually across the entire growing season and that there were no specific periods in which large numbers of fruit were lost (Fig. 2). These data suggest that there were no catastrophic periods of fruit loss triggered by extreme biotic or abiotic stresses, and therefore work was undertaken to determine whether specific environmental constraints might limit yield by limiting the availability of carbon.

In many fruit trees, such as apples, cherries, and almonds, fruit abortion occurs during specific stages of fruit development (Koukourikou-Petridou, 2003; Else *et al.*, 2004; Dal Cin *et al.*, 2009). For example, fruitlet abortion in apples may occur 5–6 weeks after full bloom or approximately 4 weeks before harvest (Arseneault and Cline, 2016). This type of fruit abortion involves the participation of hormones and is under developmental control (Bangerth, 2000). Nevertheless, higher fruit abortion may also occur due to lack of non-structural carbohydrate resources that result in higher competition between sink tissues (Gauci *et al.*, 2009; Ruan *et al.*, 2012). This type of fruit abortion is not confined to certain developmental stages of fruit and is primarily influenced by environmental inputs, source–sink interactions and anatomical characteristics (Amiard *et al.*, 2005; Demmig-Adams *et al.*, 2017). A key observation here is that fruit losses occurred throughout the growing season regardless of experimental site or growing environment (tunnel *versus* field), suggesting that resource limitations rather than developmental pathways were responsible for fruit abortion in blueberries. Such resource limitations may lead to competition for non-structural carbohydrates between fruits and new vegetation that develop concomitantly during the growing season.

Carbohydrate reserves are essential for the survival and productivity of deciduous fruit trees. They can be stored toward the end of the growing season to support developmental events that occur in spring, such as flowering and new vegetative growth (Gauci *et al.*, 2009; Da Silva *et al.*, 2014). There are also cases where carbohydrate reserves accumulate during the early stages of fruit development to support later growth of fruit (Hancock *et al.*, 2007). For most deciduous fruit trees, the prevalent form of carbohydrate reserves is starch, although many species convert starch into soluble sugars to protect against low temperatures (Jones *et al.*, 1999; Da Silva *et al.*, 2014). Here, we found that starch was not the prevalent form of carbohydrate reserve in blueberry plants during the end of the growing season. Instead, starch levels were similar and lower compared with soluble sugars in roots and stems, respectively (Fig. 3). High accumulation of soluble sugars in stems is a common feature of many deciduous trees (Da Silva *et al.*, 2014) and may be associated with bud protection against low winter temperatures (Jones *et al.*, 1999). Interestingly, starch levels did not change from autumn to winter, suggesting that the accumulation of soluble sugars in stems during winter requires the breakdown of metabolic pools other than starch to provide carbon skeletons for the formation of soluble sugars.

Analysis of non-structural carbohydrate dynamics indicated that a significant portion of non-structural carbohydrates were found in the leaves (Fig. 3), while ^13^C-labelling during fruit development revealed that much of the newly assimilated carbon was retained in leaves even after a dark period (Fig. 6). High starch levels were also found at the beginning and the end of the photoperiod in the leaves of two rabbiteye blueberry cultivars and these were not used to support fruit growth (Darnell, 1991). This is in contrast to other plant species, such as Arabidopsis, apple, grapevine and tomato, where starch accumulated in leaves during the day is hydrolysed and sugars exported to sink tissues overnight to support continued growth and development (King *et al.*, 1988; Zhou *et al.*, 2001; Gibon *et al.*, 2004; Zhu *et al.*, 2017). Several possible explanations may account for the high retention of non-structural carbohydrates in leaves. The first possibility is that the ability of blueberry sinks to utilize non-structural carbohydrates is generally low. Thus, low demand for non-structural carbohydrates (low sink strength) may lead to carbohydrate accumulation in leaves, which in turn may also result in feedback downregulation of photosynthesis (Paul and Foyer, 2001). The second possibility is that, for as yet unknown reasons, blueberries need to maintain high non-structural carbohydrate concentration in their leaves and hence they restrict carbon export to sink tissues. For example, it has been demonstrated that Arabidopsis plants increase carbohydrate levels in leaves, especially sucrose, to protect from low or chilling temperatures (Nägele and Heyer, 2013). Alternatively, newly assimilated carbon may be retained in the leaves for conversion into structural components or for repair of leaf damage.

Yield of deciduous woody perennials depends on the amount of non-structural carbohydrates allocated to the fruit compared with other sinks, and is largely determined by sink strength (Bihmidine *et al.*, 2013; Ludewig and Flügge, 2013). In general, most woody perennials allocate a high proportion of non-structural carbohydrates to fruits either during the entire developmental period (Zhang *et al.*, 2005) or at particular stages of fruit development (Hancock *et al.*, 2007). A striking finding here was that newly assimilated carbon was almost equally distributed within blueberry organs (Fig. 6), suggesting that the developing fruit is not a particularly strong sink. It is not clear whether this is due to limited utilization of non-structural carbohydrates by the fruit (sink limitation) or to limited carbohydrate resources that result in increased competition between sinks (source limitation), although current data support the latter possibility. Sink strength reflects the ability of an organ to import photoassimilates and depends on the organ’s size and its activity (Ho, 1988; Herbers and Sonnewald, 1998). In our study, the concentration of ^13^C was higher in fruit compared with other tissues (Supplementary Fig. S6), suggesting that its sink activity is also higher. However, taking into account the mass of the different organs, the sink strength of fruits was similar to that of stems and roots (Fig. 6), indicating that the main factor limiting fruit sink strength is actually the total mass of fruit. The dramatic decrease in fruit number that occurs continuously throughout the growing season, probably due to resource limitations, might account for the significant reduction in fruit sink-size. Lack of non-structural carbohydrate resources to support fruit development may result from low carbon fixation and assimilation and/or limited amount of starch reserves that eventually lead to a higher percentage of fruit abortion (Gauci *et al.*, 2009).

Taken together, these data indicate that blueberries lack a carbohydrate reserve to support fruit development and instead rely on newly assimilated carbon that must then be distributed between the major sink organs to support plant growth. The analysis reveals critical periods for resource accumulation and identifies factors related to carbohydrate partitioning that are limiting for yield. It further suggests that any environmental perturbation that negatively impacts carbon assimilation could significantly decrease the capacity of the plant to continue to support fruit development and could therefore contribute to the yield instability trait.

### Blueberry leaves exhibit biochemical and stomatal limitation to photosynthesis

Work examining carbohydrate dynamics revealed that blueberry fruit development was supported by newly acquired photoassimilates and furthermore that fruits compete for photoassimilates with other sink tissues. It was therefore important to determine the factors limiting photoassimilation within the UK and other growing environments.

In an intitial analysis, photosynthetic responses of blueberries were assessed by generating light-response and *A*–*C*_i_ curves. Under conditions of ambient CO_2_ (400 µmol mol^−1^), carbon assimilation rate reached a plateau at moderate light intensities approximating to a PPFD of 500 µmol m^−2^ s^−1^. This represents a significantly lower maximum photosynthetic rate than is seen in other commonly grown soft fruits such as strawberry (Choi *et al.*, 2016) and raspberry (Qiu *et al.*, 2017). Furthermore, whereas a very distinct plateau was observed regarding photosynthetic assimilation in blueberry leaves, strawberry leaves continue to increase their photosynthetic assimilation rate at light intensities up to at least 1000 µmol m^−2^ s^−1^ (Choi *et al.*, 2016). These data suggested that blueberry yield may be limited by photosynthetic assimilation and furthermore that year-to-year environmental variation could impose varying photosynthetic constraints leading to yield instability.

Having established that blueberry yield may be limited by photosynthetic constraints, a significant issue was to determine what specific factors were limiting photosynthesis. Under ambient CO_2_ (400 µmol mol^−1^) and 25 °C temperature, photosynthetic assimilation reached a plateau at a PPFD of approximately 500 µmol m^−2^ s^−1^ (Fig. 4A). This was coincident with the maximum electron transport rate as calculated from chlorophyll fluorescence parameters that also plateaued at an incident light intensity of approximately 500 µmol m^−2^ s^−1^ (Fig. 4D). These data are perhaps indicative of photochemical limitations to photosynthesis. However, the curve of stomatal conductance also exhibited a similar pattern to photosynthetic assimilation and electron transport rates, rising sharply to reach a maximum value at an approximate PPFD of 500 µmol m^−2^ s^−1^ (Fig. 4B). Furthermore, leaf internal CO_2_ concentration (*C*_i_) exhibited an inverse pattern, falling to a minimum value when light intensity exceeded approximately 400 µmol m^−2^ s^−1^ (Fig. 4C). These data suggest that electron transport may have been limited by electron demand required for the reductive phase of the Calvin cycle. Furthermore, the finding that the maximum electron transport rate was correlated with the minimum *C*_i_ suggests that downstream biochemical processes associated with carbon fixation may have been the ultimate limiting factor. This hypothesis was further supported by the finding that photoassimilation was significantly lower at 15 than at 25 °C (Fig. 4E) indicative of limitation by a biochemical process. More significantly, under a PPFD of 1200 µmol m^−2^ s^−1^ the increase in photosynthetic assimilation rate was almost linear in both ‘Liberty’ and ‘Duke’ leaves and assimilation was still not saturated at *C*_i_ up to approximately 1000 µmol mol^−1^. Nevertheless, whether such biochemical limitations are related to the kinetic properties of Rubisco or to enzymes other than Rubisco including those involved to the regenerative phase of the Calvin cycle requires further investigation. These data suggest that in the short term, blueberry yields might be improved by enriching the CO_2_ environment as is often the case in commercial strawberry cultivation. Longer-term breeding efforts could be directed towards enhancing stomatal density or improving Rubisco kinetics as has been suggested for cereal crops (Prins *et al.*, 2016).

Fluctuation of light irradiance may occur in the field over short timescales of seconds to minutes (Assmann and Wang, 2001) and is mainly driven by changes in cloud cover, sun angle and shading within the canopy or between adjacent plants (Pearcy, 1990; McAusland *et al.*, 2016). Data presented here indicates that in a dynamic light environment stomatal limitations to photosynthetic assimilation are further exacerbated by the slow stomatal response to changing light environments. When photosynthesis is measured in a dynamic light environment, where light intensity shifts from low to high, blueberry leaves require almost an hour to reach maximum photosynthetic rate (Fig. 5). The slow increase in photosynthetic assimilation upon shift from low to high light was mirrored by the gradual increase in stomatal conductance indicating that photosynthesis experienced stomatal limitation. This finding implies large losses in terms of cumulative carbon assimilation in dynamic light environments and emphasises the importance for securing appropriate light conditions to maximize blueberry yield. Uncoupling of carbon assimilation rate from stomatal conductance in dynamic light environments has also been demonstrated in a range of different annual species (McAusland *et al.*, 2016). In their natural habitat, blueberries grow as understorey plants with low to moderate light levels and diffuse light (Retamales and Hancock 2012). To take immediate advantage of any available light under these light-limiting conditions, one would expect that blueberries might have developed mechanisms to respond faster to sudden changes in light, such as rapid stomatal responses. Nevertheless, this is not the case; in fact, bluberries exhibit slower stomatal responses than the majority of the species examined in the work of McAusland *et al.* (2016). Faster stomatal responses to changing light may therfore be a further potential breeding goal for improving blueberry productivity.

### UK climatic conditions exhibit variation in a range that is significant for blueberry photosynthesis

Data presented in Supplementary Fig. S2 indicates that while light levels frequently exceeded a threshold of 500 µmol m^−2^ s^−1^ at the primary experimental site in Eastern Scotland throughout the growing season, there were frequent periods including many complete days when light levels remained below this threshold. Indeed, taking into account all the hours of daylight throughout the growing season, ~75% of the time light levels in polytunnels were below the threshold of 500 µmol m^−2^ s^−1^ at which blueberry photosynthesis achieves its maximum rate under ambient CO_2_ conditions. Similar results were recorded at a site further south in Herefordshire, UK, where ~50% of daylight hours were below this threshold (data not shown). Therefore, it is conceivable that yield may be limited by a different total light integral in different seasons. To confirm the importance of light for successful blueberry production, the growing environment of blueberry plants was manipulated with reflective mulches that enhance light interception (Atkinson *et al.*, 2006). Both silver and white reflective mulches increased yield dramatically through enhanced retention of fruit by blueberry plants (Fig. 7), demonstrating that light is indeed a major determinant of blueberry yield and reflective mulch treatment a potential means to achieving high yields.

Beyond an influence of light on fruit yield, temperature recording also indicated that air temperature may have a significant impact on fruit yield. At the main experimental site, temperatures fluctuated between a mean daily average of 7 °C and 20 °C (Supplementary Fig. S3), while cumulative average temperature over the entire season was approximately 8% higher in Herefordshire. These values lie within a range that will also have a significant impact on photosynthetic carbon assimilation at all light intensities (Fig. 4E).

## Conclusions

Data presented here establish that (i) blueberry fruit development is dependent on newly assimilated carbon and that blueberry plants do not store a reserve carbohydrate buffer to support fruit development in adverse environmental conditions, (ii) blueberry leaves reach a photosynthetic threshold at a light intensity of approximately 500 µmol m^−2^ s^−1^, (iii) blueberry leaf stomata are slow to respond to dynamic light environments resulting in extensive stomatal limitation in such environments, and (iv) UK growing conditions fluctuate in a range that is likely to impact photosynthetic performance. These data imply that blueberry plants are unable to compensate for low photosynthetic assimilation on days of low light intensity by increasing photosynthesis on brighter days, suggesting that blueberry yield instability may result primarily from seasonal variation in cloud cover. Many areas around the world exhibit high yield instability in their blueberry production (FAO, 2014) suggesting that the findings in the present study have much wider applicability.

The data presented here indicate several potential avenues for improving blueberry yield stability. Management options include selective pruning or the use of reflective mulches to enhance leaf light interception, growth under (heated) structures to maximize daytime temperatures to enhance photosynthesis, and the use of supplemental CO_2_. Longer term breeding options should focus on stomatal density, stomatal dynamics and downstream biochemical factors such as Rubisco kinetics.

## Supplementary data

Supplementary data are available at *JXB* online.

Fig. S1. Yield variability in blueberry production of different countries (http://www.fao.org/faostat/en/#data/QC , last accessed 9 April 2018, 2014).

Fig. S2. Photosynthetic photon flux density inside and outside a tunnel during the 2016 growing season (May to September).

Fig. S3. Mean daily temperature inside and outside a tunnel during the 2016 growing season (May to September).

Fig. S4. Relative change in the number of reproductive structures in ‘Liberty’ and ‘Duke’ plants.

Fig. S5. Light-response curves of net CO_2_ assimilation rate (*A*) of ‘Liberty’ and ‘Duke’ plants.

Fig. S6. Allocation of newly assimilated ^13^CO_2_ to individual organs at specific stages of blueberry development.

Table S1. Changes in whole organ individual soluble sugars (glucose, fructose, and sucrose) throughout the annual plant growth cycle.

Supplementary Table S1

Supplementary Figures S1-S6
