# Supplementary material for: Photosynthetic limitation as a factor influencing yield in highbush blueberries (Vaccinium corymbosum) grown in a northern European environment
Source: J Exp Bot. 2018 Mar 24;69(12):3069–80. doi: 10.1093/jxb/ery118 (PMC5972668; doi:10.1093/jxb/ery118)
Supplement: Supplementary Figures S1-S6 [file ery118_suppl_supplementary_figures_s1-s6.pdf]

Supplementary data

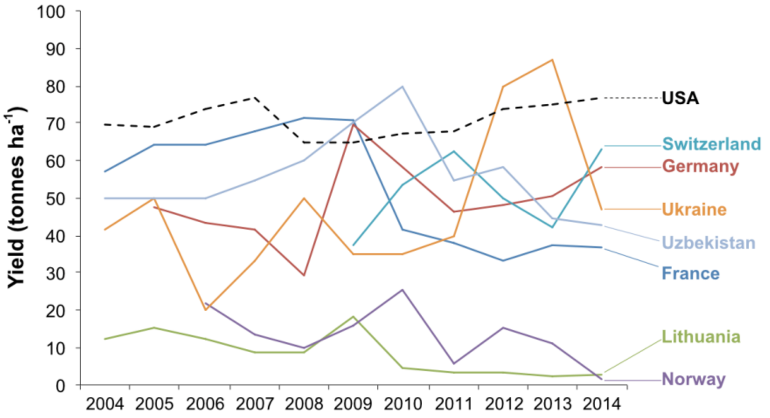

Fig. S1. Yield variability in blueberry production for different countries (http://www.fao.org/faostat/en/#data/QC, 2014). Data for USA (black dotted line) are used as a reference and indicate high and stable yield. Yield data provide information for the period 2004-2014.

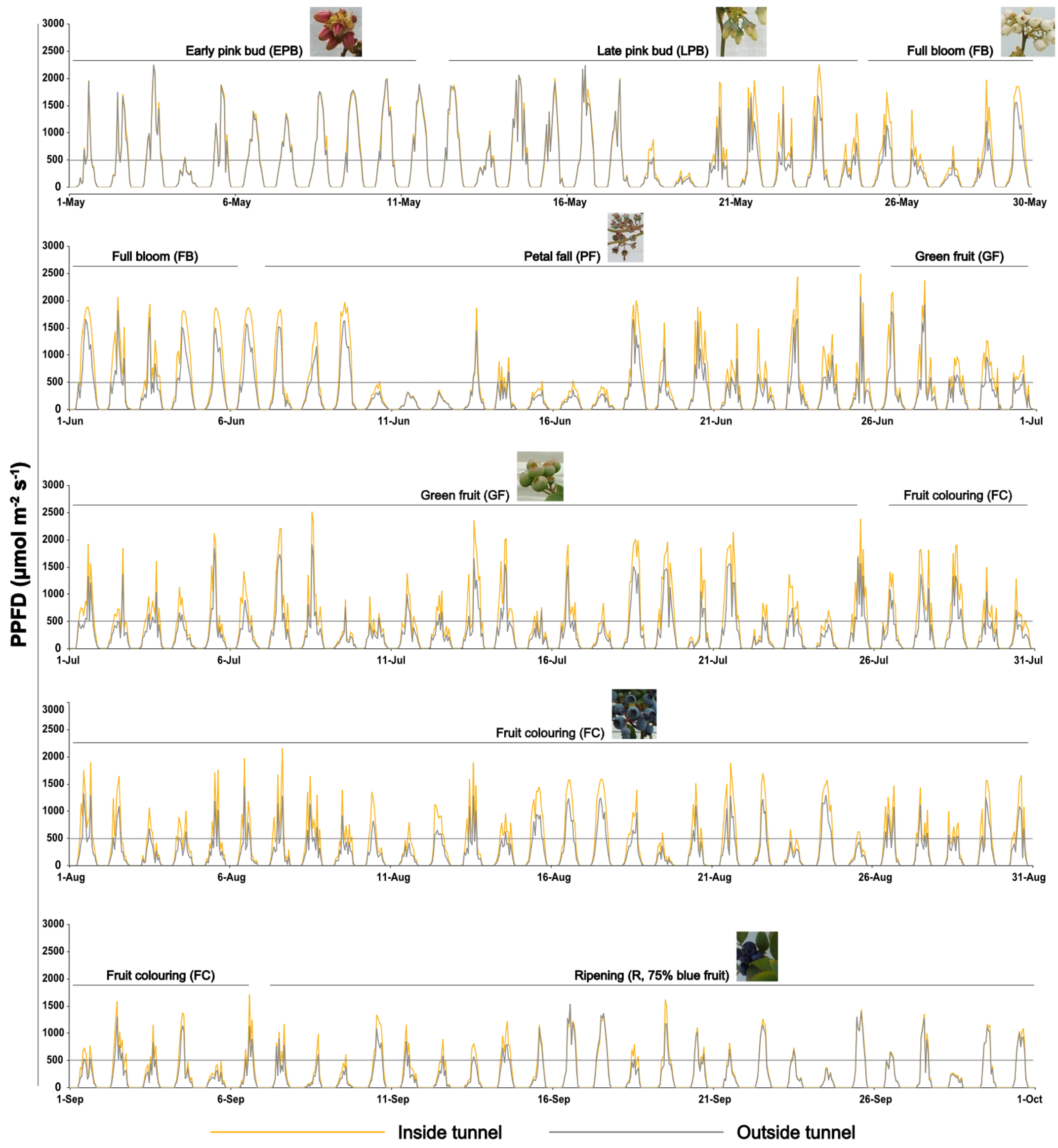

Fig. S2. Photosynthetic photon flux density inside and outside tunnel during the 2016 growing season (May to September). PPFD was recorded at hourly intervals. The approximate dates of different stages of development are indicated by horizontal lines above the chart. The horizontal black line crossing y-axis at 500 µmol m^{-2} s^{-1} PPFD indicates the irradiance where blueberry photosynthesis is saturated.

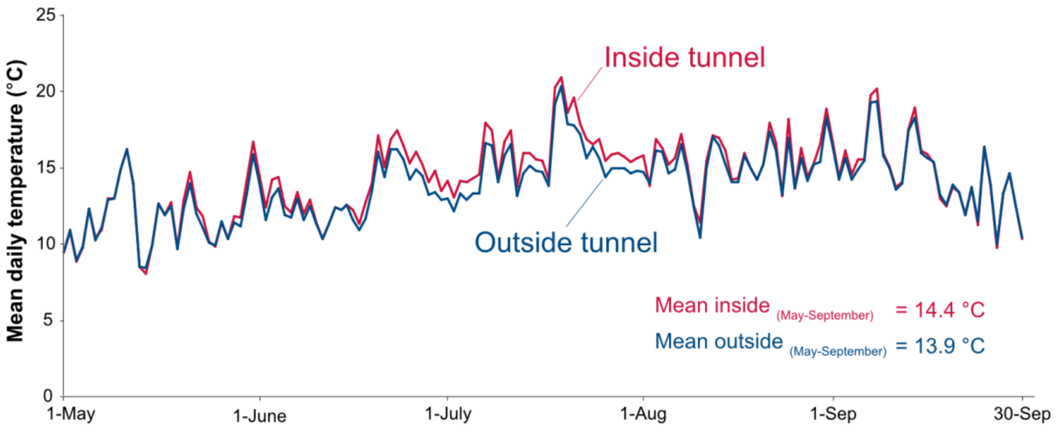

Fig. S3. Mean daily temperature inside (—) and outside (—) tunnel during the 2016 growing season (May to September).

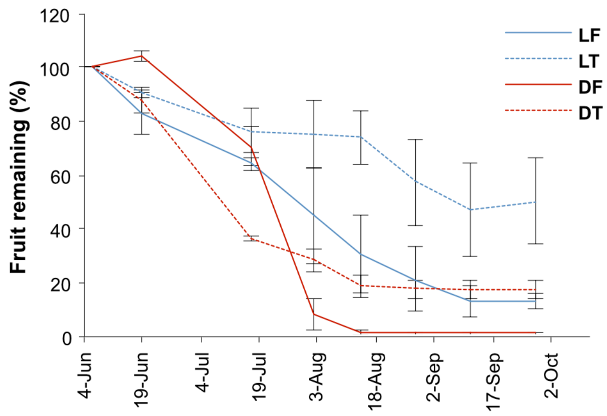

Fig. S4. Relative change in the number of reproductive structures in 'Liberty' and 'Duke' plants (n=3) during the 2017 growing season at the Invergowrie site. Plants were grown either under protective polytunnels or in the open field and the number of reproductive structures were regularly counted at different stages of development. Data represent the mean of three independent plants ± SE. LF, Liberty Field; LT, Liberty Tunnel; DF, Duke Field; DT, Duke Tunnel.

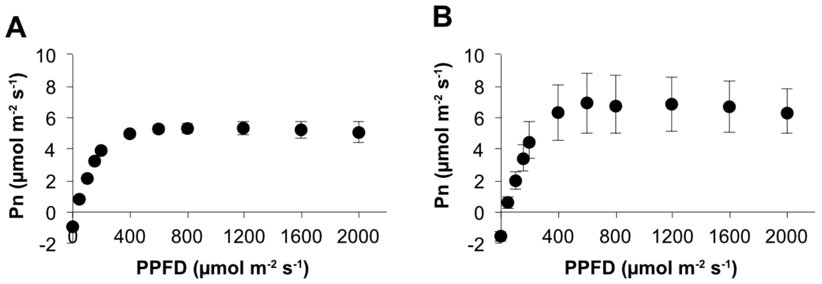

Fig. S5. Light-response curves of net CO_2 assimilation rate (*A*) of 'Liberty' (A) and 'Duke' (B) plants. All measurements were undertaken on fully developed leaves at 25˚C using a fixed cuvette CO_2 concentration of 400 μmol mol^{-1}. Data are represented as mean ± SE, n = 3.

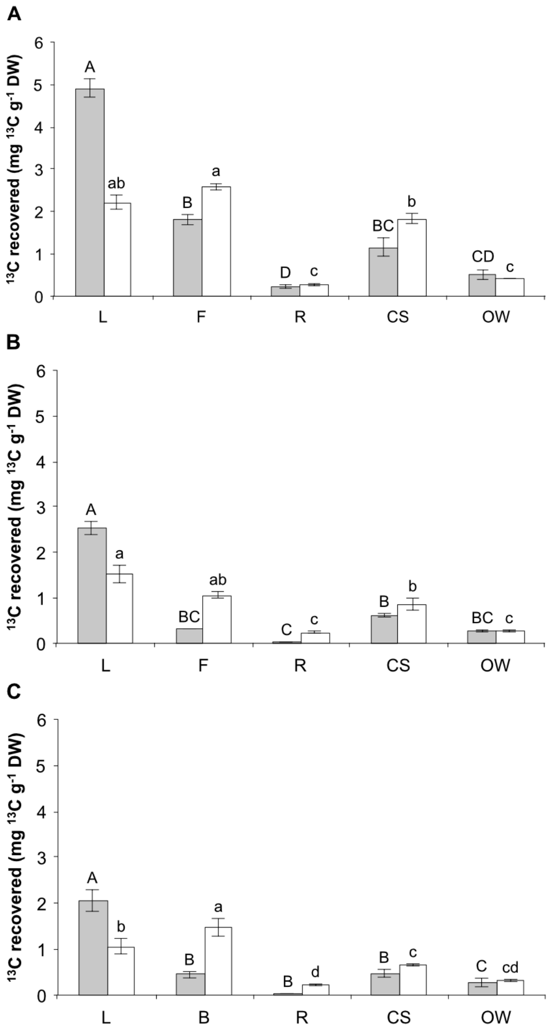

Fig. S6. Allocation of newly assimilated $^{13}CO_2$ to individual organs after 4 h (■) and 24 h (□) at fruit set (A), ripening (B) and post-harvest (C); leaf (L), fruit (F), root (R), current-year shoot (CS) and old wood stem (OW). Plants were labelled for 1 h with $^{13}CO_2$ and harvested after chasing for 4 h and 24 h. Bars represent data from 2016 growing season and are the mean value of three replicates ± SE. Data were subjected to one-way analysis of variance (ANOVA). Significant differences ($P \leq 0.05$) between means were determined using the Tukey's test. Uppercase and lowercase letters denote differences between tissues for 4 h and 24 h, respectively.
